# Entry inhibitors as arenavirus antivirals

**DOI:** 10.3389/fmicb.2024.1382953

**Published:** 2024-04-08

**Authors:** Kruthika Iyer, Zhonghao Yan, Susan R. Ross

**Affiliations:** Department of Microbiology and Immunology, University of Illinois, College of Medicine, Chicago, IL, United States

**Keywords:** arenavirus, antivirals, inhibitors, drugs, entry, fusion

## Abstract

Arenaviruses belonging to the Arenaviridae family, genus mammarenavirus, are enveloped, single-stranded RNA viruses primarily found in rodent species, that cause severe hemorrhagic fever in humans. With high mortality rates and limited treatment options, the search for effective antivirals is imperative. Current treatments, notably ribavirin and other nucleoside inhibitors, are only partially effective and have significant side effects. The high lethality and lack of treatment, coupled with the absence of vaccines for all but Junín virus, has led to the classification of these viruses as Category A pathogens by the Centers for Disease Control (CDC). This review focuses on entry inhibitors as potential therapeutics against mammarenaviruses, which include both New World and Old World arenaviruses. Various entry inhibition strategies, including small molecule inhibitors and neutralizing antibodies, have been explored through high throughput screening, genome-wide studies, and drug repurposing. Notable progress has been made in identifying molecules that target receptor binding, internalization, or fusion steps. Despite promising preclinical results, the translation of entry inhibitors to approved human therapeutics has faced challenges. Many have only been tested in *in vitro* or animal models, and a number of candidates showed efficacy only against specific arenaviruses, limiting their broader applicability. The widespread existence of arenaviruses in various rodent species and their potential for their zoonotic transmission also underscores the need for rapid development and deployment of successful pan-arenavirus therapeutics. The diverse pool of candidate molecules in the pipeline provides hope for the eventual discovery of a broadly effective arenavirus antiviral.

## 1 Introduction

New and Old World arenaviruses belong to the Arenaviridae family, genus Mammarenavirus (Grande-Pérez et al., 2016; Hallam et al., 2018; Garry, 2023). Many cause severe hemorrhagic fever when they zoonose into humans. They are classified into Old World (OWA) and New World (NWA) arenaviruses based on their geographical location and phylogenetic similarities (Salazar-Bravo et al., 2002; Charrel and de Lamballerie, 2010; Gómez et al., 2011). Lassa fever virus (LASV), an OWA found in West Africa, is the most prevalent arenavirus that infects humans, with about 100,000 to 300,000 cases annually and a mortality rate of 2–5% (Garry, 2023). Lymphocytic Choriomeningitis Virus (LCMV) is another OWA that is found in both the Eastern and Western Hemispheres; while generally not lethal, it can cause neurologic disease in immunosuppressed humans (Vilibic-Cavlek et al., 2021). On the other hand, there are five NWAs that are known to be pathogenic in humans, all found in South America: Junín virus (JUNV), Machupo virus (MACV), Sabiá virus (SABV), Guanarito virus (GTOV) and Chaparé virus (CHAV) (Sarute and Ross, 2017). The fatality rates for NWA infections can be as high as 30% (Gómez et al., 2011). Additional novel mammarenaviruses have been identified worldwide but their human pathogenic potential has not been verified (Li et al., 2015; Grobbelaar et al., 2021).

The lack of effective treatment against mammarenaviruses combined with the high mortality rate has led to these viruses being classified as Category A pathogens by the CDC, as well as on the WHO R&D Blueprint priority list (WHO R&D Blueprint priority list, 2018). The only arenavirus vaccine currently in use is Candid #1, a live attenuated JUNV vaccine strain. This vaccine has dramatically reduced the incidence of the Argentine hemorrhagic fever (AHF) when administered to at-risk individuals (Ambrosio et al., 2011; Saito et al., 2023).

Treatment for arenavirus infection is limited. The purine nucleoside analog ribavirin, a broad-spectrum antiviral used to treat a variety of RNA viruses, shows efficacy for LASV-infected patients, although with significant side effects (Salam et al., 2022). Studies on various RNA viruses showed that ribavirin may act via several mechanisms – inhibition of replication through incorporation of the analog into viral RNA, direct inhibition of the RNA-dependent RNA polymerase or inhibition of host inosine monophosphate dehydrogenase (IMPDH), RNA mutagenesis and immunomodulation (Te et al., 2007; Moreno et al., 2011). Convalescent plasma therapy and ribavirin have been used to treat JUNV infection, but due to the declining numbers of infections as a result of immunization, there are few convalescent plasma donors. Moreover, no convalescent plasma for arenavirus infections other than JUNV is available and because of the mixed efficacy and significant side effects associated with ribavirin, there is a great need for the development of new anti-arenavirus therapeutics (Enria et al., 2008; Charrel et al., 2011; Sarute and Ross, 2017, 2021).

**Figure 1:**
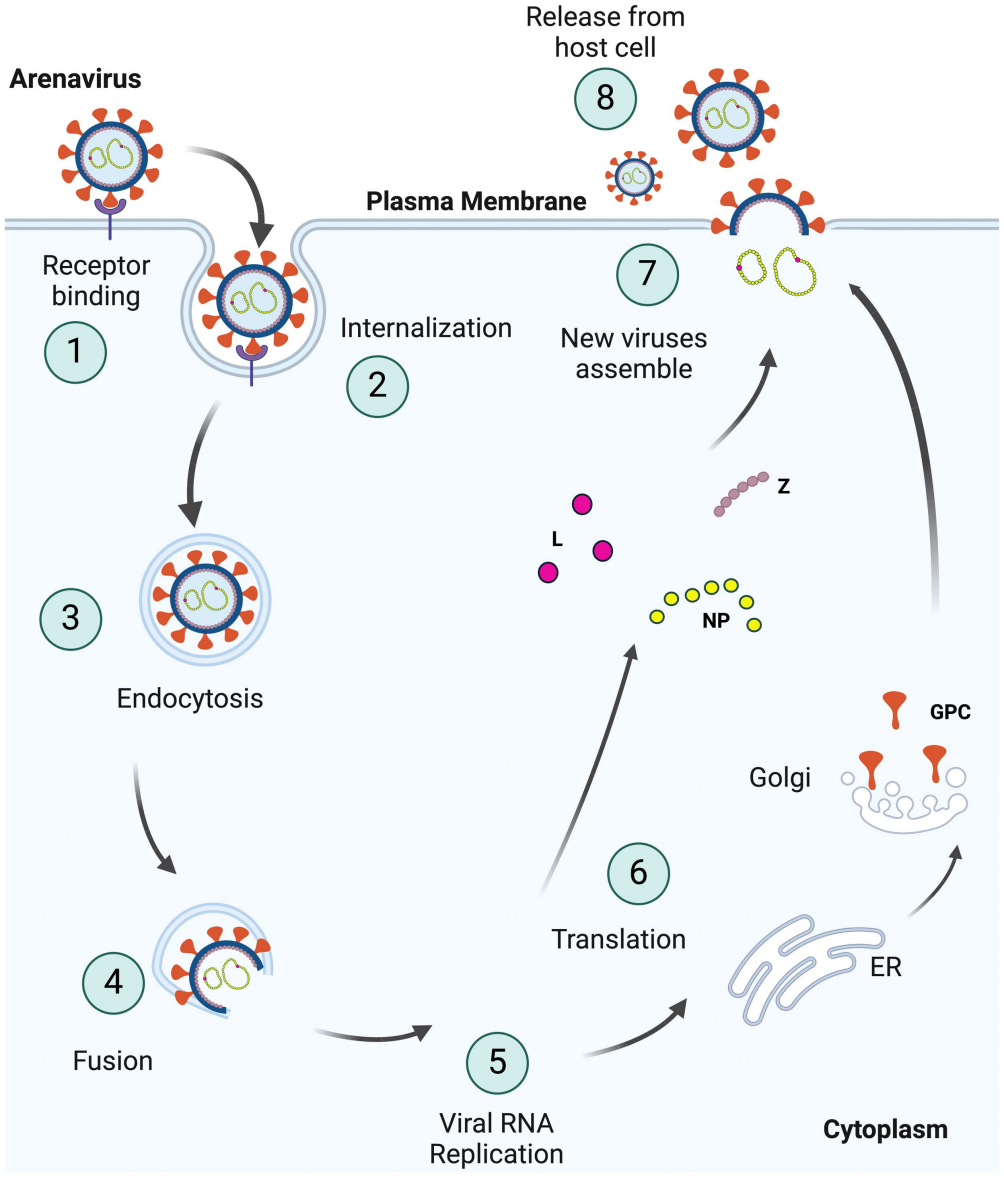
Schematic representation of the arenavirus life cycle in cells. (1) Viruses bind to specific receptors on the host cell surface to initiate invagination of the cell membrane leading to (2) internalization of the virus enclosed in a vacuole within the host plasma membrane, as part of (3) endocytosis. Internalized viruses then traffic to acidic endosomes, and the low pH environment triggers fusion (4) of the viral and host membranes. As two membranes fuse, the viral genome and proteins are released into host cytoplasm, resulting in viral (5) RNA replication and (6) translation of these viral RNAs. GPCs are processed separately in the ER and Golgi complex. The newly synthesized viral proteins (L, NP, and Z) and viral genome are (7) assembled into the virion with glycoproteins on the surface and (8) bud from the plasma membrane (Created with BioRender.com).

Entry inhibitors are good candidates for treatment, since they can slow the spread of the virus, thereby allowing the host to mount an effective immune response (Nunberg and York, 2012; Fedeli et al., 2018a). Arenaviruses use the viral glycoprotein (GP) for entry into cells. After binding to cellular receptors, virus is trafficked to a low pH compartment where fusion of the viral and cell membrane occurs (Figure 1). Entry inhibitors mostly target the receptor binding or fusion steps of entry. This review focuses on the different classes of entry inhibitors that have been developed and tested to date.

## 2 Arenavirus classification, structure and replication

The Arenaviridae family has four different genera – Mammarenavirus, Reptarenavirus, Antennavirus and Hartmanivirus (Radoshitzky et al., 2015, 2023). Reptarenaviruses and Hartmaniviruses infect snakes, while Antennaviruses are found in fish. The genus Mammarenavirus includes 42 species (Grande-Pérez et al., 2016; Hallam et al., 2018). OWAs are predominantly found on the African continent, except for LCMV which is found worldwide due to its presence in the ubiquitous species *Mus musculus* (Grande-Pérez et al., 2016; Hallam et al., 2018). LASV is the causative agent of Lassa fever and is endemic to West African countries, where its natural rodent host *Mastomys natalensis* is found (Paweska et al., 2009; Sewlall et al., 2014; Garry, 2023). LASV consists of at least seven lineages distinguished by their nucleic acid sequence and originates in various countries in West Africa. The major viruses found in Nigeria are lineage I (LP), II (803213) and III (GA391), while lineage IV (Josiah) is found in Guinea, Liberia and Sierra Leone (Bowen et al., 2000). A fifth lineage (LV) has been isolated in Mali and the Ivory Coast, a sixth lineage LVI (Kako) has been identified from *Hylomyscus pamfi* rodents in Nigeria and a seventh distinct lineage LVII from Togo (Manning et al., 2015; Olayemi et al., 2016; Whitmer et al., 2018). Lujo (Lusaka/Johannesburg) virus, another OWA believed to be carried by rodents, is the etiologic agent of Lujo hemorrhagic fever, and caused a small outbreak in Zambia and South Africa through exposure of 4 individuals to a single index case (Paweska et al., 2009; Sewlall et al., 2014). Several other OWAs have also been found in *Mastomys* and other African rodent species, although they are not associated with human disease (Saito et al., 2023).

The NWAs are distributed throughout the American continents and are divided into Clades A, B, C and A/Rec (D). The NWAs that are pathogenic in humans fall under the Clade B category and have only been found in South America (Radoshitzky et al., 2015). They include JUNV (Argentina), MACV (Bolivia), GTOV (Venezuela), SABV (Brazil) and CHAPV (Bolivia) and are named for the regions in the country in which they were discovered (Sarute and Ross, 2017; Saito et al., 2023). Argentine, Bolivian, Venezuelan, Brazilian and Chapare hemorrhagic fevers are caused by JUNV, MACV, GTOV, SABV and CHAPV, respectively (Radoshitzky et al., 2015; Sarute and Ross, 2017; Saito et al., 2023). The Clade B category includes Tacaribe virus (TCRV), originally isolated from an Artibeus bat in Trinidad, and also found in mosquitos and ticks (Downs et al., 1963; Price, 1978). It is not known to cause human disease, although it has been associated with febrile illness in lab workers (Bowen et al., 1996). Other A, B, C, and D arenaviruses, such as Whitewater Arroyo virus, Tamiami virus and Ocozocoautla de Espinosa virus, some of which may have spilled over into humans, are found in rodents throughout the Americas (Fulhorst et al., 1996; Cajimat et al., 2012; Sarute and Ross, 2017).

**Figure 2:**
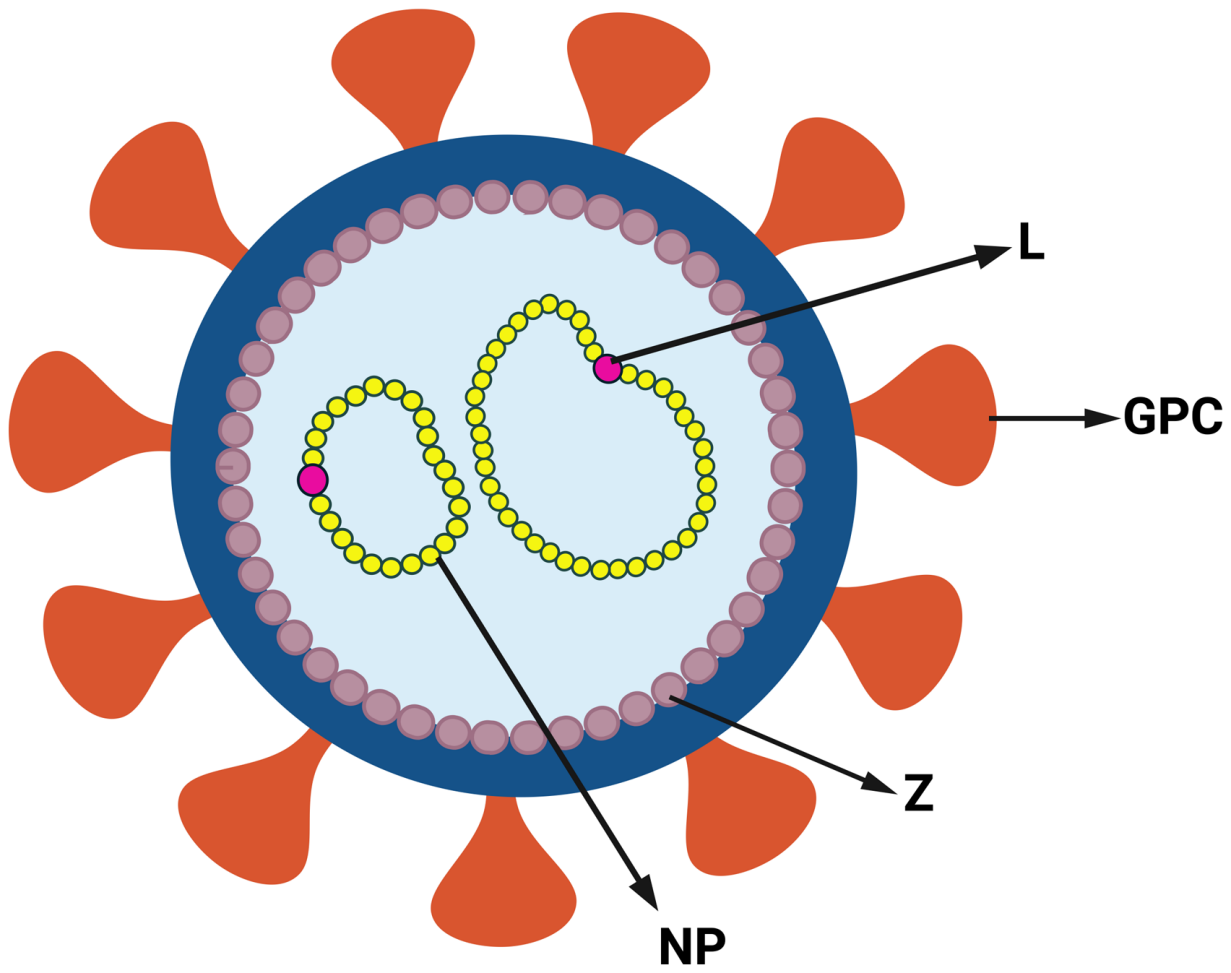
Structure of arenavirus. Simplified representation of arenavirus virion and genome. Arenaviruses have a glycoprotein complex (GPC) on the surface of the viral membrane. Inside the membrane viral genome is a capsid composed of the zinc finger matrix protein (Z). The viral genome is associated with RNA-dependent RNA polymerase (L) and nucleoprotein (NP) (Created with BioRender.com).

Arenaviruses are enveloped and have a viral genome consisting of a bisegmented, single-stranded, ambi-sense RNA (Hallam et al., 2018). The larger L segment codes for the RNA-dependent RNA polymerase (L) and the matrix protein (Z), which forms the viral capsid. The smaller S segment codes for the glycoprotein precursor (GPC) and nucleoprotein (NP), which binds the viral RNA within the capsid (Figure 2). The GPC is cleaved post-translationally into the stable signal peptide (SSP), GP1 and GP2 (Beyer et al., 2003; Rojek et al., 2008; Burri et al., 2012; Pasquato et al., 2018). Together, these form a trimer on the viral membrane; arenaviruses are unique in retaining the SSP as part of the GP complex (Burri et al., 2012; Pasquato et al., 2018). GP1 initiates viral entry by binding to cell surface receptors [(alpha-dystroglycan [alpha-DG]) for LASV, LCMV and Clade C NWAs, neuropilin for LUJV and transferrin receptor 1 (TfR1) for Clade A, B and A/Rec NWAs] (Fedeli et al., 2018a). CD164 has also been identified as an essential LCMV entry factor, as have voltage-gated calcium channels for NWAs and OWAs (Lavanya et al., 2013; Sarute and Ross, 2020; Liu et al., 2022).

After binding to cell surface receptors, arenaviruses use endocytic pathways to internalize to an acidic compartment (Fedeli et al., 2018a) (Figure 1). The SSP is essential for intracellular trafficking and proteolytic maturation of the GPC complex, as well as playing a role in pH-dependent membrane fusion. The acidic pH of the late endosomes (pH 5) triggers conformational changes in the GP that promote exposure of the fusion peptide found in GP2 and thus promotes fusion of the viral envelope with the endosomal membrane, resulting in the release into the cytoplasm of capsid containing the viral genome in complex with NP and L (Figure 1). LASV and LUJV viruses switch to endosomal receptors in the low pH compartment, lysosomal-associated membrane protein (LAMP1) and CD63, respectively, thereby triggering the fusion event; it is possible that the other arenaviruses also require as-of-yet unidentified endosomal receptors (Jae et al., 2014; Raaben et al., 2017). It was earlier thought that the GP-mediated fusion of LASV happened only at very low pH (highly acidic; <4.5) (Klewitz et al., 2007; Cosset et al., 2009). Studies later showed that in the presence of LAMP1, fusion can occur even in early endosomes, where the pH is around 5.5 (Jae et al., 2014; Hulseberg et al., 2018). The same may not be the case for LUJV, LCMV and the NWAs (Jae et al., 2014; Raaben et al., 2017).

Once in the cytoplasm, replication and translation of the viral genome begins with “snatching” of 5’ caps from host mRNAs by the virus L protein (Olschewski et al., 2020). These caps are needed for transcription initiation of the viral RNA and synthesis of positive strand viral RNA. Replication of the viral genome requires both the viral RNA polymerase (L) and NP proteins (Figure 1). All the viral proteins are synthesized by the host translation machinery. The Z, NP and L proteins are synthesized by cytosolic ribosomes. Translation of GPC occurs in the endoplasmic reticulum, where it undergoes extensive N-linked glycosylation that adds glycan molecules (Burri et al., 2012). This “glycan shield” results in evasion from neutralizing antibodies and prolonged viremia (Sommerstein et al., 2015; Koma et al., 2021). Arenaviruses GPCs undergo 2 cleavage events; cleavage of the viral glycoproteins by cellular subtilisin kexin Isozyme 1/Site 1 Protease (SKI-1/S1P) generates the outer globular GP1 domain and the membrane spanning GP2 stalk, and cleavage of the signal peptide is catalyzed by the cellular signal peptidase (SPase) (Lenz et al., 2001; Beyer et al., 2003; Rojek et al., 2008). The multifunctional Z protein controls viral expression by negatively regulating replication, transcription and translation (Cornu and de la Torre, 2001; Kranzusch and Whelan, 2011; Kang et al., 2021). Z protein further plays a role in the transport and assembly of viral components at the plasma membrane as well as the release of progeny virions from the cells (Fehling et al., 2012).

Arenaviruses successfully replicate within their host by disrupting immune responses. Suppression of the innate immune system by LASV is one of the major hallmarks of fatal and severe viral hemorrhagic fever (Mahanty et al., 2001; Geisbert and Jahrling, 2004; Russier et al., 2012; Yun and Walker, 2012). While dendritic cells and macrophages are readily infected by LASV, the virus fails to activate these cells because several viral proteins interact with cellular factors involved in the interferon response (Feldmann et al., 1996; Baize et al., 2004; Garry, 2023). Humoral immune responses are also suppressed by LASV; during pathogenic infection, neutralizing antibodies only appear several months after the acute phase (Baize et al., 2009; Saito et al., 2023). On the other hand, NWAs induce type-I IFN responses and the production of inflammatory cytokines and disease severity correlates with type-I IFN and cytokine levels (Levis et al., 1984; Huang et al., 2012, 2015); the petechiae and other sequelae of NWA hemorrhagic fever may be caused by cytokine effects on blood vessels, platelets and immune cells (Levis et al., 1985; Sarute and Ross, 2017). In the case of AHF, patients who recover from JUNV infection produce neutralizing antibodies during the acute phase of infection (Mantlo et al., 2019). Strong immune responses during the acute phase of LASV infection also correlate with recovery and survival in animal studies (Baize et al., 2009). Thus, pharmacologic suppression of virus levels at early times of infection could allow the development of protective immune responses.

Past reviews and papers have focused on anti-viral drugs and strategies that act on various steps of the arenavirus life cycle such as replication of the RNA genome, packaging and proteolytic processing of the GPC (Eunsung Mouradian and Tang, 2008; Lee et al., 2011; Pasquato et al., 2012). The present work focuses on drugs and strategies that are being tested against arenavirus entry.

## 3 Small molecule inhibitors of fusion

Low molecular weight molecules that act as drugs can affect protein interactions by forming complexes with them (Scott et al., 2016; Lu et al., 2020). As described in the preceding section, in arenavirus entry, the envelope glycoprotein plays a major role in virus entry. Once cleaved by the cellular S1P/SKI-1 protease, the GPC is cleaved to form a trimer of GP1, GP2 and SSP. The SSP forms a hairpin loop structure that spans the viral membrane and interacts with GP2 to help “sense” the acidic pH of the environment and thereby trigger viral fusion, a key step in viral entry (York and Nunberg, 2006, 2009; Agnihothram et al., 2007). Several studies reported the development of molecules that inhibit fusion by targeting the GP2-SSP interaction. One study identified a number of small molecules that inhibit infection by LASV pseudotypes using a high throughput screen of iminodiacetic acid- and pyrrolidine-based peptidomimetics as well as heterocyclic compounds (Lee et al., 2008). The lead compounds also had broad anti-viral activity against JUNV, MACV and GTOV pseudotypes. The compounds identified in this screen blocked viral entry by inhibiting pH-dependent membrane fusion in the acidic compartment after virus internalization. Three compounds, 8C1, 16G8, and 17C8, were the most potent, with an IC50 of <1 μM and also showed activity against *bona fide* LASV, JUNV and MACV (Lee et al., 2008). Photoaffinity derivatives of 16G8 confirmed that this class of inhibitors antagonize fusion by binding to the GP2-SSP interface (Shankar et al., 2016). Additional analogs of 16G8 were chemically synthesized and structure–activity relationship (SAR) studies were performed to identify molecules that were more potent and stable. They were then tested against LASV, MACV, JUNV and TCRV pseudotypes and *bona fide* viruses TCRV and LASV (Plewe et al., 2019). Several analogs showed about 100-fold greater potency than the original compounds, with a subset possessing a longer half-life as defined by the human liver metabolic half-lives. Additionally, the analogs were less potent against the JUNV vaccine strain (Candid #1) GP, possibly due to a F427I mutation found in the GP2 transmembrane domain.

Another small molecule inhibitor that has shown promise is ST-193 [1-(4-methoxyphenyl)-N-[(4-propan-2-ylphenyl)methyl]benzimidazol-5-amine], an analog of a benzimidazole derivative. Found through another high-throughput screen of 400,000 diverse small molecules using LASV pseudotyped virus, it had antiviral activity against LASV, JUNV, MACV, GTOV and SABV pseudotypes with an IC50 in the range of 0.2 to 12 nM (Larson et al., 2008). ST-193 also inhibited *bona fide* LASV, JUNV, MACV, GTOV and TCRV infection. While the mechanism of action was not elucidated, two residues in the transmembrane region of GP2 were found to confer sensitivity to the molecule. ST-193 was also shown to protect guinea pigs from lethal LASV infection at a dose of 25 mg/kg or 80 mg/kg delivered intraperitoneally, and dramatically reduced virus titers by 2-3 logs, even though animals had symptoms of fever (Cashman et al., 2011). This resulted in the generation of neutralizing antibodies by day 7 post-infection.

Along with ST-193, other chemically distinct classes of small molecules were developed by SIGA Technologies (ST-294, ST-761 and ST-161) and the Scripps Research Institute (8C1 and 17C8). All the molecules act through the SSP-G2 interface and prevent pH-induced membrane fusion (York et al., 2008; Nunberg and York, 2012). Mutations introduced in the JUNV GP2 membrane-proximal and transmembrane domains (T418N, L420T, A435I, and F438I) conferred resistance to ST-294. Homologous mutants in TCRV isolates were found to be resistant to ST-336, the parent compound of ST-294 (Bolken et al., 2006). ST-336 showed submicromolar potency against wild type TCRV as well as infection with *bona fide* MACV, GTOV, JUNV and Candid#1. ST-294 also showed anti-viral activity *in vivo*; mice injected with 100 mg/kg of drug intraperitoneally daily and infected with TCRV had increased survival (Bolken et al., 2006). Other compounds possessing a benzimidazole core, such as ST-37, were found to potently inhibit pseudotyped arenaviruses (LASV, JUNV, MACV, SABV and GTOV) at nanomolar levels (EC50 of 5 to 100 nM) (Dai et al., 2013). However, ST-193 was found to be more potent than ST-37, possessing lower EC50 values for all the pseudoviruses tested.

Subsequent studies were performed to create analogs of ST-193. One molecule, with an imidazopyridine core, showed 10-fold higher inhibitory activity against LASV pseudotyped virus when compared to ST-193, but not against other arenaviruses (Burgeson et al., 2013). LHF-535 is another analog of ST-193 that showed about 2-6 fold higher potency against a broad range of pseudotyped arenaviruses, including LASV, JUNV, MACV, TCRV, CHAPV and SABV (Madu et al., 2018). Its lack of activity against the lineage I strain of LASV and Candid#1 vaccine strain of JUNV mapped to a single amino acid in the GP2 subunit transmembrane region, similar to that seen for the 16G8 analogs (Plewe et al., 2019). However, LHF-535 protected guinea pigs against a lethal dose of lineage IV strain of LASV; intraperitoneal injection of 50 mg/kg daily resulted in 100% survival of animals and undetectable virus at day 35 post-infection, whereas all control animals died by around day 16 (Cashman et al., 2022). The inhibitor also protected mice from a lethal dose of TCRV; an oral dose of 10 mg/kg or 30 mg/kg per day decreased viral titers by 4 logs (Madu et al., 2018). Moreover, LHF-535 was tested for safety and pharmacokinetics in phase I clinical trials in healthy humans, where it exhibited a long half-life with exposures sufficient to suppress viral challenge (Amberg et al., 2022).

The success of the animal studies and phase I clinical trials underscores the importance of developing such molecules as antiviral therapies. Further compounds having a benzimidazole core were synthesized using ST-193 and LHF-535 as the lead structures. Five compounds (7d − Z, 7h − Z, 13c, 13d, and 13f) restricted pseudotyped LASV entry comparable to the parent compound LHF-535 and showed low cytotoxicity (Chen et al., 2023). The entire ST-193 class of inhibitors seems to antagonize the pH-dependent fusion required for viral entry by binding to and stabilizing the prefusion structure of the GP2-SSP complex (Nunberg and York, 2012; Shankar et al., 2016).

The same group of investigators that worked on the 16G8 molecule also chemically synthesized a series of novel heterocyclic chemical compounds through pharmacophore modeling of the piperazinone series (16G8 analogs) with the benzimidazole series (ST-193 analogs). They developed additional compounds that inhibited arenavirus fusion (Plewe et al., 2021). These molecules possessed higher stability and showed potency against pseudotyped New and Old World arenaviruses. The series included diphenyl-substituted imidazo[1,2-a]pyridines, benzimidazoles, and benzotriazoles (Plewe et al., 2021). From the same series, two additional compounds, ARN-75039, and ARN-75041 (3,6-diphenyl substituted imidazopyridines), which worked at sub-nanomolar concentrations, inhibited fusion of pseudotyped LASV, JUNV, MACV, GTOV, CHAPV, and TCRV and *bona fide* TCRV. TCRV drug resistant mutants were selected in the presence of the parent ARN compound which confirmed the mechanism of action via GP-mediated fusion. The compounds also showed potency in a mouse model of TCRV infection and had attractive drug-like properties, such as long metabolic half-lives, stability and distribution (Gowen et al., 2021). Mice treated with ARN-75039 or ARN-75041 at either 10 mg/kg or 35 mg/kg daily by oral gavage showed a 5-log decrease in viral titers at day 9 post-infection. ARN-75039 was further tested against JUNV in a guinea pig model (Westover et al., 2022). Animals orally given 60 mg/kg of the drug daily had a 3-log decrease in viral titers at day 12 post-infection. Further, treatments starting at day 2 post-infection ensured 100% survival of the animals. Finally, ARN-75039 showed a synergistic effect when it was combined with a viral polymerase inhibitor, favipiravir.

Screening of a fragment-based drug discovery library against pseudotyped LASV led to the identification of two molecules, F1920 (1-Hydroxy-2,3-dimethylbenzene) and F1965 (1(4-Chlorophenyl)-1-cyclopropanecarbonitrile), that also inhibited GP-mediated membrane fusion at high IC50s of around 50 μM (Hou et al., 2022). Both compounds also inhibited replication-competent recombinant LASV infection, albeit at even higher concentrations. Serially passaging the virus in the presence of the two molecules led to mutations which suggested that residues in the GP2 transmembrane domain played a critical role in the inhibition. The inhibitory activity of F1920 and not F1965, was extended to other arenavirus pseudotypes such as JUNV, GTOV, CHAPV, and LUJV.

Yet another compound found to inhibit GP-mediated fusion was a completely unrelated small molecule, ZCL278 [4-{3-[2-(4-bromo-2-chloro-phenoxy)-acetyl]-thioureido}-N-(4,6-dimethyl-pyrimidin-2-yl)-benzenesulfonamide] (Chou et al., 2016). ZCL278 is an inhibitor of Cdc42, a small GTPase that regulates actin polymerization (Hall, 1994; Wear et al., 2000). Surprisingly, ZCL278 did not inhibit JUNV internalization, thought to depend on actin polymerization (Martinez et al., 2009). Instead, it prevented the release of ribonucleoprotein-containing viral cores into the cytoplasm by blocking GP-mediated fusion. The inhibitor also altered the intracellular trafficking of JUNV at an IC50 of ~14 μM, such that particles were trafficked to proteolytically active lysosomal compartments. The inhibitory activity of ZCL278 was also seen with VSV, dengue virus and LCMV infection *in vitro*, as well as in mice infected with JUNV Candid#1. An advantage that ZCL278 has over other small molecules is that since it targets a cellular process, emergence of a resistant virus may be less likely. However, studies to confirm this must be performed.

Some small molecules that inhibit arenavirus entry have been discovered that do not bind to or alter GP2-SSP mediated viral fusion but instead work through disrupting critical virus-lipid interactions. One compound, adamantyl diphenyl piperazine 3.3, inhibited pseudotyped LASV through this novel mechanism (IC50 of 1.8 μM) (Wang M. K. M. et al., 2018). The endosomal protein LAMP1, which as discussed above is a critical factor for LASV fusion, binds reversibly to cholesterol (Cohen-Dvashi et al., 2016; Li and Pfeffer, 2016; Hulseberg et al., 2018). Moreover, LAMP1 binds to LASV GP in a cholesterol-dependent manner (Jae et al., 2014; Wang M. K. M. et al., 2018). A photoreactive analog of 3.3 was used in imaging studies and immunoprecipitation to investigate its mechanism of action. Inhibitor 3.3 competitively interfered with cholesterol binding to the D1 domain of LAMP1, thereby interfering with its ability to bind the LASV GP. Thus, by blocking LAMP1-GP binding, 3.3 may inhibit viral fusion and thereby entry.

From a high-throughput screen to identify Nipah virus entry inhibitors, LJ001, an aryl methyldiene rhodanine derivative, was found to block entry of several enveloped viruses at sub-micromolar concentrations, including human immunodeficiency virus (HIV), JUNV and Ebola virus (EBOV), but not non-enveloped viruses (Wolf et al., 2010). LJ001 targets and intercalates into the lipid bilayer of both the virus and cell. However, only disruption of the viral membrane inhibited virus-cell fusion. The lipid-based mechanism of LJ001 may limit the appearance of resistant viral strains; however, this remains to be tested. Similarly, a porphyrin derivative, protoporphyrin IX (PPIX) was tested against a range of enveloped viruses such influenza A virus (IAV), MACV, LASV and SARS-CoV-2 and showed an inhibitory effect at low micromolar concentrations (Lu et al., 2021). Using IAV as a test virus, it was found that PPIX binds the viral lipid bilayer and, through an unknown mechanism, prevented viral entry. Furthermore, IAV passed through 10 generations in sublethal concentrations of PPIX did not result in the decreased potency of the compound.

Sphingolipids are components of cellular membranes and metabolites of sphingolipids, such as sphingosines, are lipid second messengers involved in many cellular processes that are activated by phosphorylation (Breslow and Weissman, 2010). One study showed that sphingosine kinases (SK) are required for trafficking of EBOV to the late endolysosomal compartments where fusion occurs, and that the SK inhibitors PF-543, SK1-I and FTY-720 inhibited EBOV infection (Stewart et al., 2023). Since the trafficking of viruses to the endolysosmal compartment during arenavirus entry is similar to EBOV, these SK inhibitors also prevented pseudotyped JUNV and LASV entry at low micromolar concentrations, albeit to a lesser extent.

In summary, the diverse approaches and classes of small molecules discussed here provide a foundation for the development of antiviral therapeutics targeting arenavirus entry. These inhibitors not only demonstrate potent activity against various arenaviruses but also offer insights into the complex mechanisms involved in viral fusion, providing avenues for further research and drug discovery in the fight against these pathogenic viruses. Further, the small molecules first discovered in 2006 led to the development of drugs like ST-193 and LHF-535 that were not toxic at concentrations exhibiting anti-viral activity and which were tested in animal models and even clinical trials in 2021 and 2022 (Cashman et al., 2011, 2022; Madu et al., 2018; Westover et al., 2022). The success of these studies demonstrates the potential of these classes of inhibitors.

## 4 Natural compounds

Natural compounds, dietary supplements and herbal products are a rich source for the discovery of novel compounds that can possess antiviral properties. For example, curcumin, a major component of turmeric (*Curcuma longa*), has long been known for its antiviral properties against many viruses including HIV, hepatitis C virus and hepatitis B virus (Anggakusuma et al., 2014; Wei et al., 2017; Jennings and Parks, 2020; Ardebili et al., 2021). Use of natural compounds or their analogs may be associated with better tolerance and broader activity.

From a phytochemical series consisting of natural compounds such as flavonoids, lignans, alkaloids and quinones, five natural molecules that inhibited infection by members of the orthohantavirus, filovirus and arenavirus families were identified. Rotenone and nitrarine dihydrochloride modestly reduced pseudotyped LASV virus entry, possibly at the fusion step in the late endosomal compartment. Three additional compounds showed dramatic antiviral activity–tetrandrine, monensin sodium salt and emetine dihydrochloride; all compounds worked in the range of 2–10 μM. Using pseudotyped hantavirus to delineate the mechanism of action, the authors showed that tetrandrine affected endosomal transport and fusion, that monensin probably inhibited exit from the endosomal compartment and that emetine did not act at the entry step but probably affected a later step in replication (Mayor et al., 2021).

Yet another botanical drug library of 1058 compounds was tested against pseudotyped and recombinant LASV/VSV replication-competent viruses (Liu et al., 2021). From the library, casticin, a compound isolated from *Vitex agnus-castus* seeds, inhibited low-pH triggered GP-mediated fusion. It also inhibited the fusion of other pseudotyped arenaviruses, including JUNV, MACV, GTOV, CHAPV, LCMV, LUJV, SABV and MOPV. Passaging of LASV in the presence of casticin resulted in the emergence of a mutation of the conserved F466 in the transmembrane region of GP2. This resulted in resistance to the compound. Mutation of the same residue in NWA also conferred resistance to the compound. Bergamottin, a natural furanocoumarin found in grapefruit, was another hit from the library that inhibited the endocytic trafficking of LASV. Both compounds inhibited infection by *bona fide* LCMV at an IC50 between 0.5–5 μM, with no cytotoxicity observed at the highest tested concentration (100 μM), making these compounds promising candidates. In an independent study, capsaicin, a natural compound found in chili peppers, was found to have antiviral activity against pseudotyped LASV (EC50 – 6.5 – 15.9 μmol/L). It targeted the SSP-GP2 interface and inhibited GP-mediated membrane fusion. Mutation studies revealed that Ala25, Val431, Phe434 and Val435 in the transmembrane region of SSP-GP2 were involved in capsaicin’s antiviral activity (Tang et al., 2020). Tangeretin, a polymethoxylated flavone, is another natural compound found in citrus peels that not only antagonized pseudotyped and *bona fide* LASV infection at the fusion step, but also seven other pseudotyped arenaviruses: LUJV, MACV, Whitewater Arroyo virus (WWAV), GTOV, JUNV, SABV and CHAPV (Tang et al., 2018). The EC50s ranged between 2–12 μM depending on the virus tested.

The rich diversity of natural compounds and their potential for antiviral activity provide a valuable foundation for further research and exploration. Most of the natural compounds described here have been identified in the last 5 years and have not yet been tested in clinical settings. Understanding their cytotoxicity, physiological dose, and efficacy in *in vivo* models are crucial toward harnessing the therapeutic potential of these natural compounds in the fight against arenavirus infections.

## 5 Repurposing of drugs

Testing and validating new compounds for their clinical and therapeutic potential can take many years and is often met with failure due to low tolerance, low bioavailability, lack of efficacy, inability to scale up synthesis and safety issues. By repurposing already approved drugs, many of these challenges can be overcome (Chong and Sullivan, 2007).

A genome-wide RNAi screen revealed that the sodium-hydrogen exchanger (NHE) plays a role in LCMV infection (Panda et al., 2011). NHE is involved in macropinocytosis, a pathway used directly or indirectly by several viruses for entry, such as infectious EBOV, infectious LCMV and pseudotyped LASV and JUNV (Mercer and Helenius, 2009; Iwasaki et al., 2014; Oppliger et al., 2016). EBOV is taken up by a macropinocytosis-like mechanism and then trafficked through the early and late endosomes, followed by fusion (Nanbo et al., 2010; Saeed et al., 2010). This pathway may also be utilized by arenaviruses. It is unknown, however, at which stage the macropinocytosis pathway merges with the classical endosomal pathway.

In one study, eight drugs, including those that inhibit the NHE, were directly compared for their effect on LASV and EBOV. Five of these drugs have FDA approval (amodiaquine, clomiphene, niclosamide, sertraline, toremifene), one is licensed abroad (arbidol) and two have been evaluated in clinical trials (apilimod, for autoimmune disease and zoniporide, for ischemic injury). The drugs were all tested against pseudotyped viruses. Zoniporide, a potent NHE inhibitor, blocked infection by LCMV, LASV and EBOV (Hulseberg et al., 2019). Amiloride, another NHE inhibitor, blocked LCMV, LASV and JUNV entry. Amodiaquine, an antimalarial and niclosamide, an anthelminthic, inhibited endosome acidification and impaired LASV and EBOV entry (Hulseberg et al., 2019). Apilimod, a drug that prevents late endosome maturation by inhibiting the enzyme phosphatidylinositol-3-phosphate 5-kinase (PIKfyve – a lipid kinase important for endosome maturation), inhibited EBOV and LASV pseudotype infection (Cai et al., 2013; Nelson et al., 2017; Qiu et al., 2018; Hulseberg et al., 2019). Apilimod blocked EBOV trafficking to the late endosome, a mechanism that is common to LASV entry (Jae et al., 2014; Jae and Brummelkamp, 2015). Clomiphene, sertraline, and toremifene are cationic amphiphilic drugs (CADs) that accumulate in the late endosomes/lysosomes, causing deregulation of endolysosomal pathways (Salata et al., 2017). They showed inhibitory activity against many viruses that traffic to the endosome, such as SARS-CoV, Middle East Respiratory Syndrome (MERS)-CoV, hepatitis C virus, EBOV and LASV (Salata et al., 2017; Hulseberg et al., 2019). The IC50s for all the drugs tested with LASV were in the range of 0.05 μM to 5 μM with the exception of zoniporide (IC50 – 148.25 μM) and apilimod (IC50 – 51.05 nM). The IC50s were similar for EBOV.

Arbidol is a broad-spectrum antiviral that blocks influenza virus fusion by targeting the hemagglutinin (HA) machinery and inhibiting HA conformational rearrangements in low pH endosomes (Blaising et al., 2014; Pécheur et al., 2016; Kadam and Wilson, 2017). It also inhibited entry by pseudotyped EBOV (IC50 – 2.83 μM) and LASV (IC50 – 1.42 μM) as well as *bona fide* LASV (Hulseberg et al., 2019). The drug appears to slow the dissociation of GP1 from GP2, an early process that occurs during the fusion step. Arbidol also inhibited entry of LCMV (IC50 – 2.51 μM) and JUNV (IC50 – 0.55 μM) pseudotyped viruses and infection by TCRV (EC50 – 5.8 μM) and Pichinde arenavirus (PICV; IC50 – 7.4 μM) (Pécheur et al., 2016; Hulseberg et al., 2019; Herring et al., 2021). Additionally, arbidol in combination with amodiaquine, aripiprazole or sertraline resulted in synergistic suppression of pseudotyped LASV and JUNV infection (Herring et al., 2021).

The drugs listed above target distinct entry steps of the arenaviral infections. Further, they are orally available and are either already FDA approved or in advanced clinical trials for other diseases. This makes these molecules attractive antiviral candidates. For example, arbidol is approved for use in China and Russia against influenza (Hulseberg et al., 2019). Since the influenza and LASV arbidol IC50s are similar, the drug can potentially be used therapeutically against LASV (Hulseberg et al., 2019). A new derivative or arbidol in combination with other drugs may yield better antiviral potential.

Isavuconazole is an anti-fungal triazole that is well-tolerated in humans (Ellsworth and Ostrosky-Zeichner, 2020). It showed antiviral activity against LASV, MACV, JUNV and GTOV pseudotypes at low micromolar concentrations (Zhang et al., 2020). Using a surrogate cell–cell fusion assay, in which cells expressing viral glycoproteins fuse with those expressing cognate receptors, it was shown that isavuconazole prevented pH-dependent fusion by targeting the SSP-GP2 interface. Mutational studies showed that specific residues in the SSP and GP2 transmembrane region confer sensitivity to isavuconazole and suggested an interaction between isavuconazole and the first transmembrane region of the SSP.

High throughput screening of an FDA-approved drug library with pseudotyped LASV using a luciferase-based assay identified two compounds that blocked infection: lacidipine, a lipophilic dihydropyridine calcium antagonist (IC50 – 2.6 μM) and phenothrin, a pyrethroid found in pesticides (IC50 – 5.3 μM) (Wang P. et al., 2018). The drugs inhibited virus-cell fusion in a cell–cell fusion assay. Lacidipine was also antiviral for pseudotyped GTOV and Mopeia virus (MOPV), a non-pathogenic virus closely related to LASV (Wulff et al., 1977). Lacidipine’s calcium antagonist activity did not play a role in blocking LASV entry. Instead, mutation studies by serially passaging the virus in the presence of the drug suggested that it acts at the SSP-GP2 interface. Phenothrin inhibited entry of pseudotyped GTOV, MPOV and CHAPV but had less impact on JUNV, MACV and SABV, although what accounted for this difference was not determined. Moreover, adaptive mutations in LASV were not observed in the presence of phenothrin.

A high throughput siRNA screen against JUNV pseudoviruses found that voltage-gated calcium channels (VGCC) are critical cellular binding and entry factors for NWAs (Lavanya et al., 2013). FDA-approved channel inhibitors verapamil, nifedipine and gabapentin inhibited JUNV entry, as well as LASV and LCMV to a lesser extent (Lavanya et al., 2013; Sarute and Ross, 2020). Intravenous gabapentin treatment also decreased *in vivo* systemic JUNV Candid# 1 infection, with daily doses as low as 10 μg/g, well below the maximum tolerated dose in humans (Lavanya et al., 2013). Moreover, TCRV and JUNV Candid# 1 infection of mice heterozygous for a lethal mutation of the VGCC α1s chain were more than 2-fold more resistant to infection and had 2-fold greater inhibition by gabapentin treatment compared to wild type mice (Sarute and Ross, 2020).

The calcium-activated potassium channel, KCa3.1 is another druggable target and marker for cancer and vascular diseases and potent, specific KCa3.1 inhibitors have been tested both *in vitro* and *in vivo* for sickle cell anemia, malaria and stroke (Ataga et al., 2008; Chou et al., 2008; Tubman et al., 2015; Staal et al., 2017). Clotrimazole is one of the best characterized and specific inhibitors of KCa3.1 (Ishii et al., 1997). However, it has a high toxicity (Alvarez et al., 1992). Analogs lacking this toxicity, TRAM-34 and senicapoc were developed and were shown to have antiviral activity against pseudotyped arenaviruses (IC50s 11 – 25 nM) (Wulff et al., 2000; Stocker et al., 2003; Chou et al., 2008; Torriani et al., 2019). TRAM-34 inhibited the fusion step of LCMV, TCRV, MACV, GTOV and LUJV but not LASV (Torriani et al., 2019). However, the mechanism of action was independent of KCa3.1 inhibition.

From a library of 102 clinical compounds, losmapimod was identified as an inhibitor of LASV pseudotype infection (EC50 of 3.68 μM) (Zhang et al., 2019). It also inhibited infectious LASV (lineage IV). The compound, a p38 mitogen-activated protein kinase (MAPK) inhibitor, was initially developed to treat chronic obstructive pulmonary disease (COPD) and cardiovascular diseases (Norman, 2015; Segalés et al., 2016). An aryl heteroaryl bis-carboxyamide derivative, it targets the SSP-GP2 interaction and prevents virus-cell fusion, similar to ST-193 and lacidipine (York et al., 2008; Nunberg and York, 2012; Wang P. et al., 2018). However, unlike ST-193, losmapimod showed activity against multiple LASV strains, including lineage I, suggesting a slightly different mechanism of action.

In addition to alpha-DG, LASV uses alternative entry receptors such as the receptor tyrosine kinase (RTK) Axl (Fedeli et al., 2018b) and hepatocyte growth factor receptor (HGFR) (Oppliger et al., 2016; Fedeli et al., 2020). R428 is a potent inhibitor of Axl RTK phosphorylation and shows activity against breast cancer metastasis in animal models (Holland et al., 2010; Shen et al., 2018). EMD, an inhibitor of HGFR, also has anti-tumor properties (Bladt et al., 2013; Oppliger et al., 2016). Both molecules inhibited recombinant LASV entry by disturbing the trafficking of virus-containing vesicles, causing them to accumulate near the cell membrane, with IC50s in the low micromolar range (Oppliger et al., 2016; Fedeli et al., 2020).

Other alternative receptors used by LASV and LCMV are C-type lectins, such as dendritic cell-specific ICAM-3-grabbing nonintegrins (DC-SIGN and L-SIGN) and liver and lymph node sinusoidal endothelial calcium-dependent lectin (LSECtin) (Shimojima et al., 2012; Shimojima and Kawaoka, 2012; Goncalves et al., 2013). Lectins interact with mannose residues on virus glycoproteins via their carbohydrate recognition domain (Feinberg et al., 2001; Mitchell et al., 2001). Treatment of cells with the anti-DC-SIGN monoclonal antibody DC28 reduced binding of chimeric LASV particles to immature monocyte-derived dendritic cells (Goncalves et al., 2013). Mannan sugars also blocked virus binding, implicating the use of C-type lectins in infection (Shimojima et al., 2012; Goncalves et al., 2013). C-type lectins C-SIGN and L-SIGN are also utilized by JUNV and infection is blocked by mannans and their respective antibodies (Martinez et al., 2013). Griffithsin is a lectin that shows great antiviral activity against HIV-1 and SARS-CoV-2, among other viruses (Lusvarghi and Bewley, 2016; Cai et al., 2020). Therefore, it has a potential to be used against arenaviruses that depend on lectins for entry.

Chlorpromazine, imipramine, desipramine, and amitriptyline are drugs used to treat various mental illnesses, but also cause lipid accumulation (Lange et al., 2012; Miller et al., 2012). Additionally. chlorpromazine inhibits clathrin-mediated endocytosis (Martinez et al., 2007). Chlorpromazine inhibited several arenavirus pseudotypes, such as CHAPV and JUNV, but showed the most significant effect on LUJV and LASV (Tani et al., 2014). Imipramine, desipramine, and amitriptyline only inhibited LUJV and LASV and not other arenaviruses. U18666A, an inhibitor of cholesterol synthesis, showed a similar effect on LUJV and LASV (Cenedella, 2009; Tani et al., 2014). This suggests that lipids play a major role in arenavirus entry and that drugs affecting lipid and cholesterol synthesis pathway could be tested for their antiviral activities.

Iron post-transcriptionally regulates TfR1 mRNA levels via iron regulatory proteins (IRPs) that bind to the iron responsive elements (IREs) in the 3’ untranslated region (UTR) (Casey et al., 1989; Ponka and Lok, 1999). When iron levels are low, IRPs bound to the IREs in the 3’UTR prevent degradation of its mRNA. In contrast, increased iron levels lead to rapid degradation of TfR1 mRNA. Iron supplementation in cell culture was shown to decrease pseudotyped JUNV and MACV but not LASV infection (Radoshitzky et al., 2007). Therefore, therapeutic iron supplementation could be considered in conjunction with other treatments.

Overall, repurposing approved drugs offers a promising strategy for the rapid development of antiviral therapies against arenaviruses. Further, when drugs target cellular proteins or processes or are used in combination, the risk of emergence of resistant viral strains remains low. However, studies to look into this aspect needs to be performed. For example, various strains of influenza virus resistant to arbidol have been reported (Blaising et al., 2014). The physiological concentrations of drugs required for inhibiting arenaviruses also needs to be determined for many of these candidates. However, leveraging existing pharmacological knowledge can potentially accelerate the timeline for clinical implementation.

## 6 Antibodies

Antibodies are of special interest in the effort to combat viral infections because of their high degree of specificity and role in adaptive immunity (Rajewsky, 2019). Vaccines inducing the production of neutralizing antibodies that block virus entry can fail due to the high rate of genetic mutations that occur in viruses. However, antibodies are being engineered for both prophylactic and therapeutic purposes, not only in viral infections but also in cancer and autoimmune diseases (Brekke and Sandlie, 2003). The isolation and generation of broadly neutralizing antibodies has become of special interest, especially against viruses such as HIV, influenza virus and LASV, which lack effective treatments, and because these viruses are able to effectively evade the immune system (Walker and Burton, 2018). The confirmed effectiveness of convalescent patient serum in controlling Junín infection that was used prior to the development of the Candid#1 vaccine suggests that this is likely to be feasible for arenaviruses (Maiztegui et al., 1979).

Several studies have examined neutralizing antibodies against the LASV GPC (Sommerstein et al., 2015). Development of neutralizing antibodies that act against all LASV lineages, including the newly emerging strains such as LV to LVII, is important (Manning et al., 2015; Olayemi et al., 2016; Whitmer et al., 2018; Buck et al., 2022). One study characterized the epitopes recognized by 16 neutralizing monoclonal antibodies (mAbs) isolated from Lassa fever survivors. The epitopes, found on different parts of GPC, were divided into four groups: GPC-A, GPC-B, GPC-C, and GP1-A. Recognition by three of the antibodies only needed the GP1 subunit and therefore were classified under the group GP1-A. The antibodies in the remaining three groups bound the GP complex and not the GP1 or GP2 subunit alone (Robinson et al., 2016). In particular, these antibodies recognized epitopes that formed a bridge between GP1 and GP2 or needed both subunits in the pre-fusion GP complex. Further, most antibodies bound to the GPC-B group of epitopes and consequently, LASV GP in conjugation with a GPC-B mAb 37.7H, was structurally characterized by crystallography (IC50 – 6.92 μg/mL) (Hastie et al., 2017, 2019). This analysis revealed the regions of LASV GP that enable efficient infection, including the binding sites for alpha-DG and LAMP1, and the glycan shield on the fusion peptide and showed that conformational changes in GP1 occur during pH-mediated fusion. Finally, using pseudotyped LASV, it was shown that mAb 37.7H prevented virus-cell fusion, likely by inhibiting the pH-mediated conformational change in GP1.

A cocktail consisting of three of the above antibodies, 8.9F, 12.1F and 37.2D, labeled as Arevirumab-3, neutralized all lineages of LASV and was effective in blocking infection in non-human primates (Mire et al., 2017; Cross et al., 2019; Li et al., 2022). The animals received 15 mg/kg of each mAb and all treated animals survived a LASV challenge. The protection was observed even with a dose as low as 1.5 mg/kg/antibody, which is lower than the US FDA approved Ebola REGN-EB3 mAb cocktail (Inmazeb) (50 mg/kg of each mAb) (Cross et al., 2019; Markham, 2021). 8.9F blocked GP/alpha-dystroglycan interaction and thus inhibited attachment of virus to the cell. 12.1F entered the host cell with the virus and probably locked the GP in a prefusion state thereby preventing receptor switching to LAMP1. 37.2D also seemed to lock the GP complex in a prefusion state, but prevented the dissociation of GP1 and GP2. Together, the three antibodies could break through the GPC glycan shield, which poses a significant barrier to neutralizing antibody production as well potentially preventing the generation of escape mutants (Robinson et al., 2016; Li et al., 2022).

However, many of these antibodies were not effective against the lineage I LASV strain. The 18.5C GPC-B mAb was engineered to neutralize lineage I LASV by addition of one or two arginine residues into the complementarity-determining regions (CDR) responsible for antigen binding (Hastie et al., 2019). The engineered 18.5C mAb had a neutralization IC50 of 2 – 8 nM against *in vitro* infection with recombinant lineage I LASV. Another LASV GPC-B mAb, M28, was engineered to neutralize LCMV. It bound to a pre-fusion epitope of the LCMV GP complex and was able to protect mice both prophylactically and therapeutically from virus challenge (Moon-Walker et al., 2023).

To find antibodies with more potent neutralization of the viral glycoprotein, the GPC-A group of epitopes were also structurally characterized. Cryoelectron microscopy and X-ray crystallography of GPC-A antibodies 25.10C and 36.1F, respectively, in conjugation with GP was done. The 25.10C antibody from this group was highly potent against infection by different lineages of pseudotyped LASV, whereas antibody 36.1F was lineage IV specific in an *in vitro* assay (IC50s in the submicromolar range) (Robinson et al., 2016). This may be because the third CDR of the antibody heavy chain (H3) of 36.1F contacts residues N74, E76 and M96 of GP1, which are not well-conserved across the LASV lineages, whereas the H3 of 25.10C CDR contacts only E76, which is conserved. The 36.1F structure in complex with GP indicated that it binds to a loop in GP1 that would block access to LAMP1, while the 25.10C mAb recognized the fusion loop of GP2. In the presence of LAMP1, fusion can occur in early endosomes where the pH is around 5.5 (Jae et al., 2014; Hulseberg et al., 2018). Thus, 36.1F may inhibit GP-LAMP1 association in an early endosomal compartment before the pH drops to <4.5. On the other hand, 25.10C may inhibit LASV infection in a LAMP-1 and pH-independent manner in later low pH endosomal compartments, possibly by occupying the GP2 fusion loop and blocking viral-membrane host fusion (Enriquez et al., 2022).

Neutralizing mAbs to Junín virus have also been identified that protect guinea pigs and nonhuman primates from infection (Zeitlin et al., 2016, 2021). Immunization-elicited antibodies against NWAs that block binding of their GPs to human TfR1 (hTfR1) have also been isolated and could be used therapeutically (Amanat et al., 2020; Ng et al., 2022).

A different approach to inhibit virus entry has taken advantage of the well-characterized structural features of hTfR1 and its interaction with NWA GPs (Abraham et al., 2010). It is well-established that NWAs and transferrin bind to different sites on the receptor (Radoshitzky et al., 2008). In an attempt to target a host protein instead of a viral protein, the mAb ch128.1/IgG3, which binds an apical region of hTfR1 and blocks the virus binding site, was utilized (Ng et al., 2006; Helguera et al., 2012). This interaction did not compete with transferrin binding but efficiently inhibited the entry of pseudotyped JUNV, GTOV, CHAV, SABV, and MACV at subnanomolar concentrations. LASV was not inhibited. The pathogenic NWAs do not use mouse TfR1 for entry (Radoshitzky et al., 2008), so to test the *in vivo* efficacy of this model, 3-week-old transgenic mice that express hTfR1 were used for infection with the Romero strain of JUNV (Hickerson et al., 2020). This small animal model allowed the testing of an IgG1 version of ch128.1. *In vivo* studies showed that JUNV infection was prevented by intraperitoneal injection of ch128.1/IgG1 (2 or 3 antibody injections of 400 μg each time) (Hickerson et al., 2022). This antibody minimally affected transferrin binding or ferritin uptake.

Docking analyses showed that the residues of hTfR1 that bind with ch128.1 are used by MACV GP1 as well. Competition between the ch128.1 and MACV GP1 for hTfR1 binding was demonstrated by flow cytometry and bio-layer interferometry (Helguera et al., 2012; Hickerson et al., 2022). A murine mAb, OKT9, also targets hTfR1 and sterically blocks binding of GP1, with IC50s of subnanomolar concentrations, thereby inhibiting entry of clade B NWAs but not LASV (Ferrero et al., 2022). Since it is believed that all clade B pathogenic NWAs use the same binding site on hTfR1, such monoclonals could be used therapeutically to treat different NWA infections. One group successfully created a TfR1 immunoadhesin (molecules that are engineered to encode viral cellular receptors fused to the Fc region of antibodies) (Cohen-Dvashi et al., 2020). This molecule, called “Arenacept,” bound to and neutralized the GPs from JUNV, GTOV, MACV, and SABV (IC50 – 0.4 – 3.4 μg/mL) and inhibited *bona fide* JUNV and MACV infection in cell culture.

The ongoing advancements in antibody research offer a diversified and dynamic arsenal against viral infections. From broadly neutralizing antibodies targeting viral glycoproteins to those directed against host factors, these findings contribute to the expanding toolkit for therapeutic interventions against arenaviruses.

## 7 Discussion

**Figure 3:**
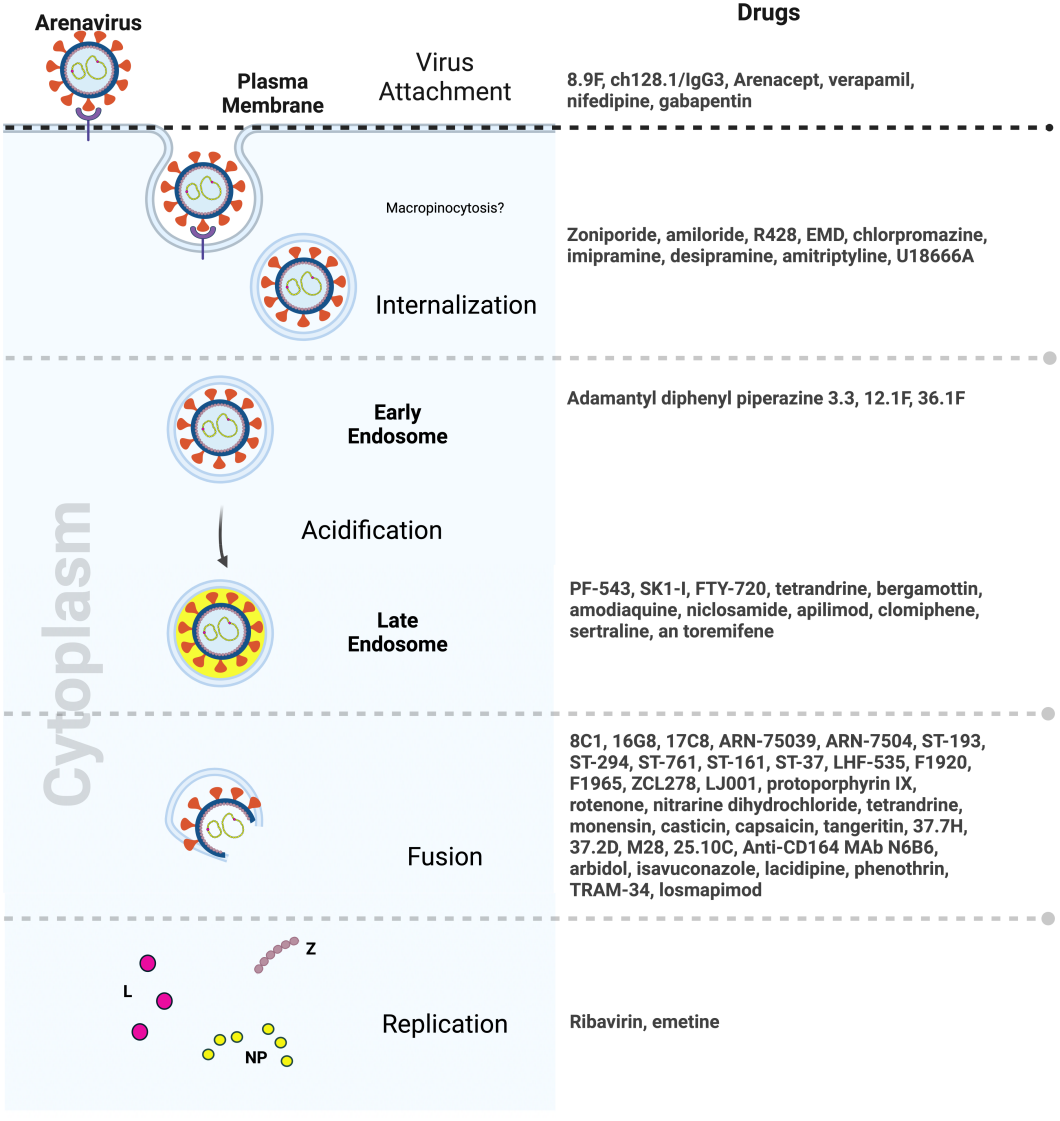
Drugs targeting distinct steps of arenavirus entry (Created with BioRender.com).

Entry inhibitors which hinder viral spread are potential therapies that will allow the host time to mount an effective immune response. Small molecule inhibitors targeting arenavirus entry have emerged as a promising area of drug development. To date, most inhibitors primarily focus on disrupting key viral protein interactions, particularly the GP2-SSP interface, thereby impeding the pH-dependent membrane fusion step essential for viral entry. Notably, compounds such as 8C1, 16G8, and 17C8, as well as the ST-193 class of inhibitors, including ST-37, LHF-535, and their analogs, showed significant potential in inhibiting viral entry by multiple arenaviruses. Moreover, advancement of some of these compounds in animal testing as well as early clinical trials in the last few years signals hope for an arenavirus anti-viral very soon (Larson et al., 2008; Lee et al., 2008; Dai et al., 2013; Madu et al., 2018; Cashman et al., 2022) (Figure 3).

The exploration of natural compounds, dietary supplements, and herbal products as potential sources for antiviral discovery has also unveiled candidates with favorable and diverse antiviral properties. Compounds such as tetrandrine, monensin sodium salt, and emetine dihydrochloride exhibit significant antiviral activity, affecting different stages of the viral life cycle (Mayor et al., 2021). Capsaicin from chili peppers and tangeretin from citrus peels that target the SSP-GP2 interface and fusion steps in arenavirus entry, respectively, have also emerged as potential antivirals (Tang et al., 2018, 2020) (Figure 3). While these natural compounds hold promise for clinical significance, their precise mechanisms of action remain unclear, and their interactions with viral glycoproteins, cellular receptors, or membranes have yet to be fully elucidated. Additionally, their effectiveness in *in vivo* models remains to be tested.

Repurposing already approved drugs perhaps offers a more efficient avenue for addressing lethal and emerging viral infections, including those caused by arenaviruses. This approach capitalizes on existing knowledge about drug safety, bioavailability, and tolerability. Several FDA-approved drugs, including zoniporide, amiloride, and amodiaquine, have demonstrated effectiveness against arenaviruses by targeting NHE, part of the micropinocytosis pathway, thereby disrupting various stages of the viral entry process (Hulseberg et al., 2019). The broad-spectrum antiviral drug arbidol, known for its efficacy against influenza, has exhibited inhibitory effects on arenaviruses such as LASV, JUNV, and others. Moreover, since arbidol is already approved for use in humans against influenza in some countries, its clinical use for arenavirus infections in humans could be tested (Blaising et al., 2014; Hulseberg et al., 2019). Lastly, clinical drugs used in mental illnesses, such as chlorpromazine, imipramine, desipramine, and amitriptyline, as well as the cholesterol synthesis inhibitor U18666A, have shown inhibitory effects on arenaviruses, emphasizing the role of lipids in the viral entry process (Tani et al., 2014) (Figure 3). Similarly, gabapentinoids that inhibit VGCCs and are widely used to treat neuropathic pain and epilepsy in humans, showed promise in animal models in reducing NWA infection (Lavanya et al., 2013; Sarute and Ross, 2020).

Antibodies play a pivotal role in the battle against viral infections, offering specificity and adaptive immunity. Convalescent patient serum has historically demonstrated effectiveness in controlling JUNV infection, supporting the feasibility of antibody-based interventions for NWAs and OWAs. Antibodies engineered for prophylactic and therapeutic purposes have shown promise. For example, the LASV GP has emerged as a primary target for neutralizing antibodies (Sommerstein et al., 2015; Robinson et al., 2016; Buck et al., 2022; Enriquez et al., 2022). However, the high genetic mutation rates in viral strains and the emergence of antibody-resistant viral mutants can pose challenges to the development of pan-viral antibodies. Therefore, targeting host proteins offers an exciting alternative. One of the best-known examples of drugs targeting a host protein is maraviroc which targets the host HIV co-receptor CCR5 (Woollard and Kanmogne, 2015). In case of NWAs, targeting the human transferrin receptor presents an alternative avenue for therapeutic intervention. The successful use of antibodies like ch128.1/IgG1 against JUNV in transgenic mice expressing human transferrin receptor highlights the potential for host-targeted antibodies *in vivo* (Hickerson et al., 2020, 2022) (Figure 3). It was also interesting to note that the antibody concentrations required to elicit antiviral responses was much lower than those of other small molecules and drugs. This is important in a clinical setting where toxicity can be a major concern.

It is of paramount importance to find an antiviral as the pathogenic arenaviruses such as LASV and JUNV cause high fatality rates when they infect humans. A significant number of inhibitors were developed to initially combat LASV and then tested against other arenaviruses. Although drug-resistance mutants were generated in the presence of some of the molecules listed above, these experiments were performed to better understand the compound’s mechanism of action. In depth studies to understand the rate of mutation of viruses during treatment, a major concern while targeting viral proteins, is needed. There is also a high possibility of zoonosis of arenaviruses that are circulating in their natural reservoirs which could be pathogenic in humans. Studies will need to look at the broad efficacy of all compounds against newly emerging arenaviruses. However, with many promising leads, it is likely that some of the candidates can soon be used in a clinical setting.

## Author contributions

KI: Conceptualization, Formal analysis, Investigation, Writing – original draft. ZY: Conceptualization, Formal analysis, Investigation, Writing – review & editing. SR: Conceptualization, Writing – review & editing, Funding acquisition, Project administration, Supervision.
